# Single intra-articular injection of high molecular weight hyaluronic acid for hip osteoarthritis

**DOI:** 10.1007/s10195-015-0381-8

**Published:** 2015-10-08

**Authors:** Fabrizio Rivera

**Affiliations:** Department of Orthopedic Trauma, SS Annunziata Hospital, Via Ospedali 14, 12038 Savigliano (CN), Italy

**Keywords:** Hip, Viscosupplementation, Hyaluronic acid

## Abstract

**Background:**

Intra-articular (IA) injection of hyaluronic acid (HA) into the hip joint appears to be safe and well tolerated but only a small number of randomized clinical trials in humans has been published. The objective of this prospective study was to evaluate the efficacy and safety of a single IA injection of high-molecular-weight (2800 kDa) HA (Coxarthrum) for hip osteoarthritis.

**Materials and methods:**

All patients received a single IA administration of 2.5 % sodium hyaluronate (75 mg/3 mL) of high molecular weight. Fluoroscopy requires an iodized contrast medium (iopamidol, 1 ml) which highlights the capsule before administering HA. Patients were evaluated before IA injection (T0), after 3 months, after 6 months and after 1 year from injection. Results were evaluated by the Brief Pain Inventory (BPI II), Harris Hip Score and a visual analog scale of pain (pain VAS). All treated patients were considered for statistical analysis.

**Results:**

Two hundred seven patients were included at T0. The mean age was 67 years (range 46–81). Regarding BPI severity score, changes in pain between T0 and the three following visits were statistically highly significant (*p* < 0.001). Changes in pain score compared to the previous visit were statistically significant for the worst pain in the second quarter post-intervention (*p* = 0.037) and for mean pain in the second semester post-intervention (*p* = 0.043) The evolution of the Harris Hip Score was statistically highly significant (*p* < 0.001) between T0 and the following visits (T0 + 3 months, T0 + 6 months and T0 + 12 months); after a significant change between T0 and T0 + 3 months, the score remained stable. The evolution of the pain VAS showed a statistically highly significant improvement (*p* < 0.001) between T0 and T0 + 3 months; thereafter it remained stable from the first quarter post-intervention. No serious adverse event was noted; 12 cases (0.5 %) of pain associated with transient synovitis are noteworthy.

**Conclusion:**

This study shows that a single IA injection of Coxarthrum is effective from the third month and that the results are stable or continue to improve up to 1 year.

**Level of evidence:**

IV.

## Introduction

Osteoarthritis has a very high prevalence globally. It is a source of pain and deterioration of joint function, with important socioeconomic consequences. The related pain is poorly evaluated by doctors, who underestimate its intensity when the pain is reported by the patient as being high, and overestimate it when it is reported as being weak [1]. The incidence of hip arthritis is increasing with age and is estimated at between 47.3 (95 % confidence interval [CI] 27.8–66.8) [1] and 88/100 000 patient-years (95 % CI 65–101) [2]. Hip pain is reported by 19.2 % (95 % CI 17.9–20.6) of people aged 65 years and older. Less than half (48 %) of the symptomatic respondents had unilateral problems affecting one hip or knee joint only [1].

Optimal management of osteoarthritis requires a combination of non-pharmacological and pharmacological modalities. Among the latter are injections of intra-articular (IA) hyaluronic acid (HA), first isolated in 1934 by Karl Meyer in the vitreous humor; the first human clinical use of IA HA in the treatment of knee arthritis was made in 1975, and the first trials date back to 1980 [1]. HA is a polysaccharide macromolecule, a glycosaminoglycan of high molecular weight (MW) composed of repetitions of disaccharides of glucuronic acid and *N*-acetylglucosamine; it is a constituent of synovial fluid in normal and osteoarthritic joints and is synthesized by chondrocytes and synoviocytes [2]. HA has complex biological properties that could explain its analgesic effects (anti-inflammatory by inhibiting the formation and release of prostaglandin, immunomodulatory in situ), irrespective of its mechanical action on the joint fluid. The concentration of HA in an arthritic joint has been found to decrease to 50–33 % of normal levels, and includes a reduction in molecular size. Molecular interaction has also been observed, with a consequent decrease in elasticity and viscosity of the synovial fluid [3].

HA may be useful in patients with knee or hip osteoarthritis. The symptomatic benefit is delayed in comparison with that of intra-articular injections of corticosteroids, but it is prolonged. The IA injections of HA are widely used and recommended in existing guidelines as a useful therapeutic modality to treat patients with knee osteoarthritis; there is less experimental evidence of efficacy for hip arthritis than for knee arthritis [4].

IA injection of HA into the hip joint appears to be safe and well tolerated [5] but only a small number of randomized clinical trials in humans has been published [6–9].

Data from a meta-analysis in knee arthritis suggested that the heterogeneity between trials might be due to the higher MW products having greater efficacy. Indeed, HA preparations may broadly be classified according to their MW and formulation type: solutions of low MW (500–1200 kDa), solutions of high MW (6000 kDa), cross-linked HA and solutions of non-animal stabilized HA (NASHA) [10–12].

As a consequence, the objective of this prospective study was to evaluate the efficacy and safety of IA injection of a single dose of high MW (2800 kDa) HA (75 mg/3 mL) (Coxarthrum, LCA Pharmaceutical, Chartres, France) for hip osteoarthritis.

## Materials and methods

The study protocol was approved according to the modalities planned by Ethical Committee. We conducted a single-center, prospective, unblinded study. After baseline (T0), patients were to be reviewed at 3, 6 and 12 months. Inclusion criteria were age more than 40 years, mono- or bilateral hip arthritis with X-ray proof of at least partially preserved joint space (Kellgren–Lawrence stage 2–3 [10]), good or full joint mobility, and hip disease persisting for at least 3 months. Patients were excluded from the study where they had severe arthritis for which it was no longer possible to recognize radiographic joint space (Kellgren–Lawrence stage 4), had inflammatory, autoimmune and septic disease (rheumatoid arthritis, connective tissue disease, osteomyelitis), or had surgical indication for hip arthroplasty. All patients received a single injection of a single administration of 2.5 % sodium hyaluronate (75 mg/3 mL) of high MW (2800 kDa) (Coxarthrum). This is a sterile, viscoelastic, transparent, homogeneous preparation composed of purified HA, without any avian protein; it is not cross-linked by a chemical agent, which limits as much as possible the risks of allergic and cytotoxic reactions. Injections were performed by fluoroscopic guidance. Fluoroscopy requires an iodized contrast medium (iopamidol, 1 ml) which highlights the capsule before administering HA. Patients were evaluated before IA injection (T0), after 3 months, after 6 months and after 1 year from injection. The first endpoint was the score on the Modified Brief Pain Inventory (BPI II) comprising (1) a score of pain severity (BPI severity score) rated between zero and 10 and measuring the pain which the subjects felt before the present visit (the worst pain, the lightest pain, the mean pain) and the pain now, that is to say the pain felt during the visit; (2) an impact score (BPI interference score) rated between zero and 10, describing disturbances of social life (work, sleep and mood); (3) an overall impact score adding the previous score and four other items (activities in general, ability to walk, relationships with others, the enjoyment of life). Another criterion of evaluation was the Harris Hip Score whose range is from zero to 100 with points distributed within four areas: “pain” domain, maximum 44 points; “function” domain, maximum 47 points; “range of motion” domain, maximum 5 points; “no deformity” domain, maximum 4 points. Finally, a visual analog scale (VAS) of pain (pain VAS), scored from zero to 10, also allowed judging the effectiveness of IA HA.

All treated patients were considered for statistical analysis, which was performed in SAS^®^ software (version 9.2). At each study time (T0, T0 + 3 months, T0 + 6 months and T0 + 12 months), mean, standard deviation and median endpoints were calculated. For the same endpoint, comparisons were made at different study times using Student’s *t* test for paired samples. Results were considered statistically significant for values of *p* < 0.05.

## Results

Two hundred seven patients were included at T0. One hundred twenty-six were women (61 %) and eighty-one were men (49 %). The mean age was 67 years (range 46–81). Mean body mass index (kg/m^2^) was 22.8 (range 18.8–29.9). Radiological evaluation of osteoarthritis showed a Kellgren–Lawrence stage 2 in 83 (40.1 %) patients and a Kellgren–Lawrence stage 3 in 124 (59.9 %) patients. The number of included patients who completed the questionnaires in the various planned visits gradually decreased over time; however, data from three-quarters of patients were still available after 6 months, and data from over half the patients after 1 year. The data of pain VAS were less available than those of the BPI II questionnaires and the Harris Hip Score (Table 1).

**Table 1:** Number of patients completing the questionnaires at the various visits

	No. of patients available at			
	T0	T0 + 3 months	T0 + 6 months	T0 + 12 months
BPI II questionnaire (severity and interference)	207	207	150	121
Harris Hip Score	207	207	150	121
VAS of pain	165	176	128	104

**Fig. 1:**
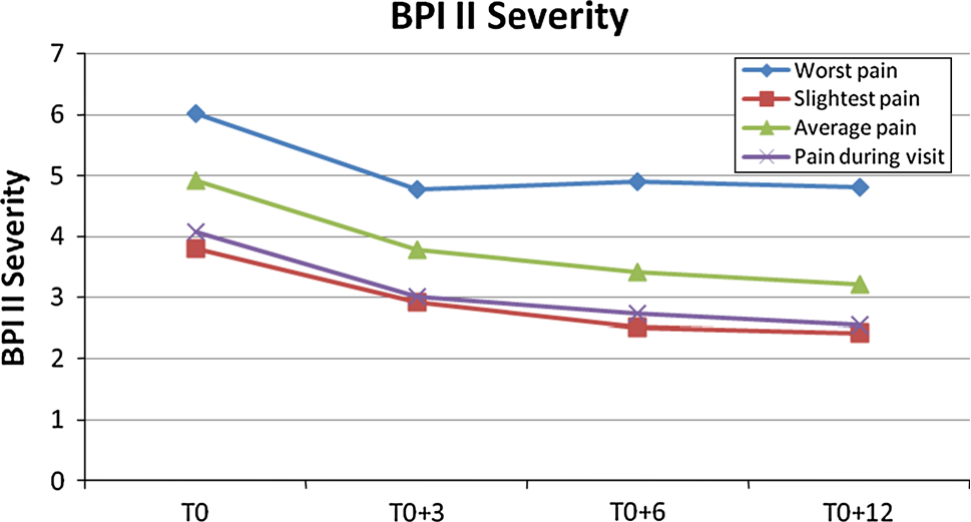
Evolution of BPI Severity Score during follow-up

Regarding the BPI severity score, changes in pain between T0 and the three following visits were statistically highly significant (*p* < 0.001). Changes in pain score compared to the previous visit were statistically significant for the worst pain in the second quarter post-intervention (*p* = 0.037) and for mean pain in the second semester post-intervention (*p* = 0.043) (Table 2). Changes in pain severity (BPI severity score) are shown in Fig. 1. Note the parallelism of the curves, although the intensity of the worst pain is virtually unchanged from T0 + 3 months onwards.

**Table 2:** Evolution of patients’ pain (BPI II severity score)

	Mean pain rating (out of 10) ± SD			
	Before and at T0	Between T0 and T0 + 3 months	Between T0 + 3 months and T0 + 6 months	Between T0 + 6 months and T0 + 12 months
Worst pain	6.03 (1.51)	4.78 (1.95)*	4.90 (2.16)*^,**†**^	4.80 (2.00)*
Slightest pain	3.80 (1.92)	2.91 (1.68)*	2.52 (1.61)*	2.42 (1.43)*
Mean pain	4.93 (1.49)	3.78 (1.64)*	3.42 (1.68)*	3.22 (1.57)*^,**†**^
Pain during visit	4.07 (2.04)	3.00 (1.94)*	2.73 (1.98)*	2.55 (1.63)*

* Statistically highly significant (*p* < 0.001) compared with T0

^**†**^Statistically significant (*p* < 0.05) compared with the previous visit

The evolution of the BPI interference score, describing disturbances of social life, measured between T0 and the three following visits, was statistically highly significant (*p* < 0.001) for the three items describing disturbances of social life (work, sleep and mood). The evolution of the BPI interference score measured against the previous visit was also highly significant (*p* < 0.001) from the second quarter post-intervention for sleep and for the second semester post-intervention for mood. It was significant (*p* < 0.05) concerning professional activities for the second semester post-intervention (Table 3).

**Table 3:** Evolution of the BPI interference score describing disturbances of social life

	Mean rating (out of 10) ± SD			
	Before and at T0	Between T0 and T0 + 3 months	Between T0 + 3 months and T0 + 6 months	Between T0 + 6 months and T0 + 12 months
Professional activities	4.44 (2.15)	3.42 (1.94)*	3.26 (2.45)*	2.59 (1.97)*^,**†**^
Sleep	3.80 (2.23)	2.59 (1.76)*	2.01 (1.78)*^,√^	1.31 (1.17)*^,**√**^
Mood	4.11 (2.18)	3.14 (2.12)*	2.79 (2.45)*	1.83 (1.61)*^,**√**^

* Statistically highly significant (*p* < 0.001) compared with T0

^**†**^ Statistically significant (*p* < 0.05) compared with previous visit

^√^ Statistically highly significant (*p* < 0.001) compared with previous time

The evolution of the BPI interference overall score between T0 and the three following visits was statistically highly significant (*p* < 0.001). The evolution of the BPI interference overall score measured against the previous visit was statistically significant in the second quarter post-intervention (*p* < 0.01) and statistically highly significant during the second semester post-intervention (*p* < 0.001) (Table 4).

**Table 4:** Evolution of the BPI interference overall score at the different visits

	Before and at T0	Between T0 and T0 + 3 months	Between T0 + 3 months and T0 + 6 months	Between T0 + 6 months and T0 + 12 months
Mean (±SD)	30.40 (13.65)	22.81 (11.92)*	19.83 (13.72)*^,**†**^	14.17 (9.78)*^,**√**^
Median	30	20	20.5	10

* Statistically highly significant (*p* < 0.001) compared with T0

^**†**^ Statistically significant (*p* < 0.01) compared with previous time

^**√**^ Statistically highly significant (*p* < 0.001) compared with previous visit

**Fig. 2:**
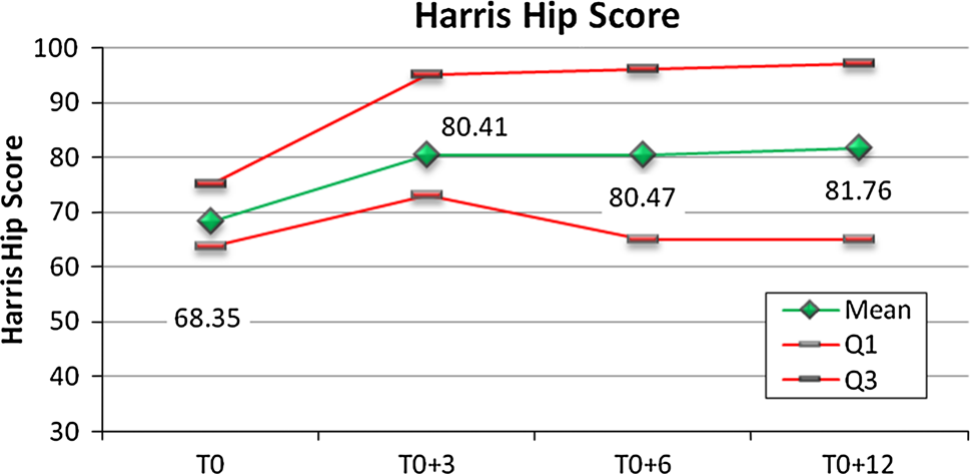
Evolution of Harris Hip Score during follow-up

The evolution of the Harris Hip Score was statistically highly significant (*p* < 0.001) between T0 and the following visits (T0 + 3 months, T0 + 6 months and T0 + 12 months); after a significant change between T0 and T0 + 3 months, the score remained stable (Fig. 2).

As for the evolution of the Harris Hip Score, the evolution of the pain VAS showed a statistically highly significant improvement (*p* < 0.001) between T0 and T0 + 3 months; thereafter it remained stable from the first quarter post-intervention.

No serious adverse event was noted; 12 cases (0.5 %) of pain associated with transient synovitis (during 24 h) are noteworthy.

## Discussion

To evaluate the efficacy and safety of a single IA of HA (75 mg/3 mL) of high MW (2800 kDa) (Coxarthrum) for hip osteoarthritis pain management, we included in our study two hundred seven patients. In this unblinded prospective study, changes in all outcome measures were significantly in favor of a single IA injection, whether it was the BPI severity score, the BPI interference score, the Harris Hip Score or the pain VAS. This trend was very clear from T0 + 3 months; then the results remained stable or continued to improve on all these criteria.

Only a small number of scientific papers containing statistically significant results about hip IA injection of HA are available in the literature despite the hip being the second most common site of arthritis.

Conrozier and colleagues [11] retrospectively evaluated a group of 56 patients with severe or moderate hip osteoarthritis after one or two IA administrations of high MW HA. At 90 days follow-up, 58.9 % of the patients reported a benefit of the infiltration treatment.

A prospective double-blind study compared the effect of high MW and low MW HA, together with a placebo. In this study 59 patients were evaluated at time intervals of 1, 3 and 6 months after the first infiltration. Similarly to our study, improvement of scores was noted at 1 month and remained significant up to 6 months in both groups compared to the placebo group (*p* < 0.001). No significant differences were observed between the results obtained in the two study groups treated with the different HA molecules [12].

Berg and Olsson [13] studied a group of 31 patients with hip osteoarthritis at 2 weeks and 3 months follow-up after a single administration of non-animal, stabilized HA (NASHA). Three months after administration, there was a statistically significant 68 % improvement in symptoms (*p* < 0.007).

Colen and colleagues [5] evaluated the efficacy of hip viscosupplementation by analysing the results of 16 trials with a total of 509 patients, with evidence levels varying from I to IV, and using various types of preparations. Notwithstanding the relatively low level of evidence in the trials, the authors concluded that viscosupplementation may be an alternative therapy for treating coxarthrosis. Intra-articular infiltration has proved to be safe and well-tolerated therapy. However, the authors state the need for trials on a larger number of people in order to avoid having to consider HA infiltration in the hip as an extremely selective choice that depends on the experience of the operator. The same group of authors [14] reported that 51 % of the patients had not undergone surgery 3 years after viscosupplementation, after evaluation of a group of 120 patients who were candidates for surgical treatment with a total hip arthroplasty.

To overcome the problem of selectivity and operator-dependent approach to hip viscosupplementation, a clear identification of whether or not the patient is suitable for HA infiltration treatment in the hip is mandatory. Although studies of variability in the efficacy of HA infiltration therapy in the hip compared to the gravity of the hip arthritis have not yet been carried out, it can reasonably be assumed, as reported by knee viscosupplementation studies, that the possibilities of efficacy and duration of the beneficial effects of the treatment are inversely proportional to the gravity of the disease [15]. For this reason selection criteria for the candidate patient are vital to obtain pain relief in cases of hip arthritis. These selection criteria consist of hip pain for at least 3 weeks, X-ray proof of at least partially-preserved joint space, and good or full joint mobility. Hip viscosupplementation can be used as an alternative to or in combination with drugs for pain control. This type of approach to viscosupplementation therapy does not correspond to the inclusion criteria reported in Van den Bekerom and colleagues’ study [14]. Considering hyaluronic acid as a pain therapy, using its beneficial effects on cartilage due to both the pharmacological and the physical properties of the molecule [16–18], then the use of injection in cases of low or medium degrees of hip arthritis is mandatory. For this reason, patient candidates for hip arthroplasty were excluded for our study.

Recently, to clarify some aspects of viscosupplementation treatment, a review of the literature confirmed that IA HA is an effective treatment for mild to moderate osteoarthritis but it is not an alternative to surgery in advanced cartilage degeneration [19].

Fluoroscopic guidance is one of the possible radiological guidances when performing IA hip injection. Due to the narrow IA space, performing a “blind” hip IA injection is not recommended [20]. Ultrasound guidance does not need contrast media and can also be repeated without causing problems of radiation load for the patient or operator, but exposure to radiation during fluoroscopy is minimal and there is no difference in the speed of IA injection between the two techniques when performed by experts. The choice between ultrasound or fluoroscopy is based on the experience of the operator in using both methods [20]. However, when IA injection is performed under fluoroscopy, the amount of radiopaque contrast agent must be as low as possible to avoid viscosupplement dilution [19].

In our experience, we observed 12 cases of pain associated with transient synovitis after IA injection. As reported in the literature [21–24], transient synovitis correlated with the reaction to a foreign body is a minor complication with an incidence of between 5 and 10 %. This adverse reaction normally resolves in 24–48 h following infiltration. without long-term clinical effects.

Further studies are needed on unresolved hip viscosupplementation issues including cost–effectiveness of therapy, relation between molecular weight and effectiveness, and how to best incorporate viscosupplementation into an arthritis therapy algorithm. Our experience proved the efficacy of IA HA injection for hip arthritis treatment.

A single dose of HA (75 mg/3 mL) of high MW (2800 kDa) is proving to be safe and effective for pain control in patients with hip arthritis (Kellgren–Lawrence stages 2 and 3) before indications for hip arthroplasty. Viscosupplementation is effective from the third month and the results are stable or continue to improve up to 1 year.
